# Laser scanner and UAV digital photogrammetry as support tools for cosmic-ray muon radiography applications: an archaeological case study from Italy

**DOI:** 10.1038/s41598-023-46661-4

**Published:** 2023-11-15

**Authors:** Tommaso Beni, Diletta Borselli, Lorenzo Bonechi, Luca Lombardi, Sandro Gonzi, Laura Melelli, Maria Angela Turchetti, Livio Fanò, Raffaello D’Alessandro, Giovanni Gigli, Nicola Casagli

**Affiliations:** 1 Department of Earth Sciences, University of Florence, Via Giorgio La Pira 4, 50121 Florence, Italy; 2 National Institute for Nuclear Physics INFN, Division of Florence, Via Bruno Rossi 1, 50019 Sesto Fiorentino, Italy; 3 Department of Physics and Geology, University of Perugia, Via Alessandro Pascoli, 06123 Perugia, Italy; 4 Department of Physics and Astronomy, University of Florence, Via Giovanni Sansone 1, 50019 Sesto Fiorentino, Italy; 5 Ministry of Culture Regional Directorate of Museum Umbria, Necropolis of Palazzone, Perugia, Italy; 6 National Institute for Nuclear Physics INFN, Division of Perugia, Via Alessandro Pascoli, 06123 Perugia, Italy

**Keywords:** Imaging techniques, Physics, Solid Earth sciences, Geomorphology, Geophysics

## Abstract

The use of light detection and ranging technologies, i.e. terrestrial laser scanner (TLS), airborne laser scanner (ALS) and mobile laser scanner (MLS), together with the unmanned aerial vehicles digital photogrammetry (UAV-DP) and satellite data are proving to be fundamental tools to carry out reliable muographic measurement campaigns. The main purpose of this paper is to propose a workflow to correctly plan and exploit these types of data for muon radiography aims. To this end, a real case study is presented: searching for hidden tombs in the Etruscan necropolis of Palazzone (Umbria, Italy). A high-resolution digital elevation model (DEM) and three-dimensional models of the ground surface/sub-surface of the study area were created by merging data obtained using different survey methods to achieve the most accurate three-dimensional environment. Indeed, the simulated muon flux transmission used to infer relative transmission values, and the estimated density distribution, depends on the reliability of the three-dimensional reconstructed ground surface model. The aim of this study is to provide knowledge on the use of TLS and UAV-DP data and GPS-acquired points within the transmission-based muography process and how these data could improve or worsen the muon imaging results. Moreover, this study confirmed that muography applications require a multidisciplinary approach.

## Introduction

In the last 20 years, many improvements have been made in the field of remote sensing (RS) close-range survey methodologies applied to several research fields, including engineering geology, natural hazard assessment, geomorphology, seismology, glaciology and structural geology^1^. The term “close-range” refers to all measurement situations in which the distance between the studied target and the scanner/camera/detector is approximately 300 m^2–4^. Unmanned aerial vehicles (UAV), also known as drones, equipped with instruments such as optical and/or thermal cameras, tighter with light detection and ranging (LiDAR) instruments such as terrestrial laser scanners (TLS), airborne laser scanners (ALS), and mobile laser scanners (MLS), are widely used in many research fields, particularly geosciences worldwide^1,5,6^. This is because of their low cost, acquisition speed, and ease of application in dangerous and inaccessible environments. Many studies have confirmed the affordability and reliability of remote sensing techniques using UAV for landslide mapping and characterization^7,8^, multitemporal fluvial geomorphological evolution^9^, cultural heritage risk monitoring, protection, and conservation^6,10^, mapping tools for structural geology^11^, and post-fire vegetation survey campaigns^12^. Also LiDAR survey methods, i.e. TLS, ALS, and MLS have been successfully used in several applications, including the geo-structural analysis of rock mass discontinuities, rock fall susceptibility scenarios, landslide monitoring^7,13–16^, and monitoring of cultural heritage^10,17,18^. Many studies have confirmed the validity and reliability of merging LiDAR and UAV-DP methods (primarily to reduce the presence of shadow zones), such as three-dimensional rock mass discontinuity characterization^2^, river channel mapping^19^, and documentation and preservation of cultural heritage^20^.

The main advantage of using RS close-range technologies is the ability to obtain data with high spatial and temporal resolution that can be easily integrated with other ground-based techniques^21^. The aim of the presented study is to show that the latter sentence is also valid for integration between RS methods and muon radiography (or muography).

Muography is an innovative, non-invasive, geophysical imaging technique that measures the naturally produced muon flux and enables the internal imaging of a wide range of targets i.e., the studied object, and with an appropriate data analysis procedure allows to estimate the density distribution of the domain under study for the various lines of sight (LoS) of the used detector^22,23^. Muons are low-interacting particles that, thanks to their high energy (kinetic energies varying from GeV to TeV) and large mass, can pass through hundreds of meters of rock before stopping their run, and this is the reason why they are employed in radiographic measurements. These particles are characterized by an average lifetime of 2.2 µs and a mass approximately 200 times that of the electron^24^. Muons are continuously produced in the Earth’s upper atmospheric layers (~ 15 to 17 km) mainly by decays of pions and kaons, which are particles generated by the collision between primary cosmic rays and the atmospheric nuclei of oxygen and nitrogen. The muon flux at sea level varies with respect to the altazimuth angles, i.e. zenith (*θ*) and azimuth (*φ*), mainly depending on the zenith ∝ *cos*^*n*^* θ*, with *n* = 2 for energies ∼ 3 GeV^24^. The natural muon flux is measured along user-defined directions and then compared to the simulated flux obtained using the same detector setting and a reconstructed three-dimensional environment with the aim of creating transmission and density maps^23,25–27^. Due to the natural downward run direction of muons (from the upper atmosphere to the Earth's surface), the employed muon detector must be positioned beneath or in close proximity to the target being studied^28^. The obtained density maps allow the identification of contrasting density regions that can be interpreted in several ways, depending on the studied target and purpose of the muography campaign survey^29^.

Several studies have demonstrated the maturity of muon radiography. Among these, muography has been successfully used for internal imaging of volcanoes^30–32^, in mining sites^26,33–36^, for civil and environmental engineering issues^27,37^, for archaeological and cultural heritage non-destructive surveys^38–41^. Since the 1990s, there has been a growing focus on integrating and comparing muography technique with other well-established geophysical survey methods, particularly gravimetry^42^. Notable studies by Nishiyama et al.^43^ and Lesparre et al.^44^ have discussed the correlation between gravity inversion results and muographic surveys for imaging the internal density of volcanoes. Oláh et al.^45^ demonstrated the utility of muography as a support tool for ground surface deformation monitoring techniques, enhancing the modeling of eruptive processes for forecasting purposes. In addition, significant advancements have been made in the field of mining survey measurements over the past decade^46^. Recently, detailed reviews and guidelines on this topic have been published^22,35,47–49^ but to the best of the authors knowledge, no detailed papers or reviews are available on the presented topic.

**Figure 1:**
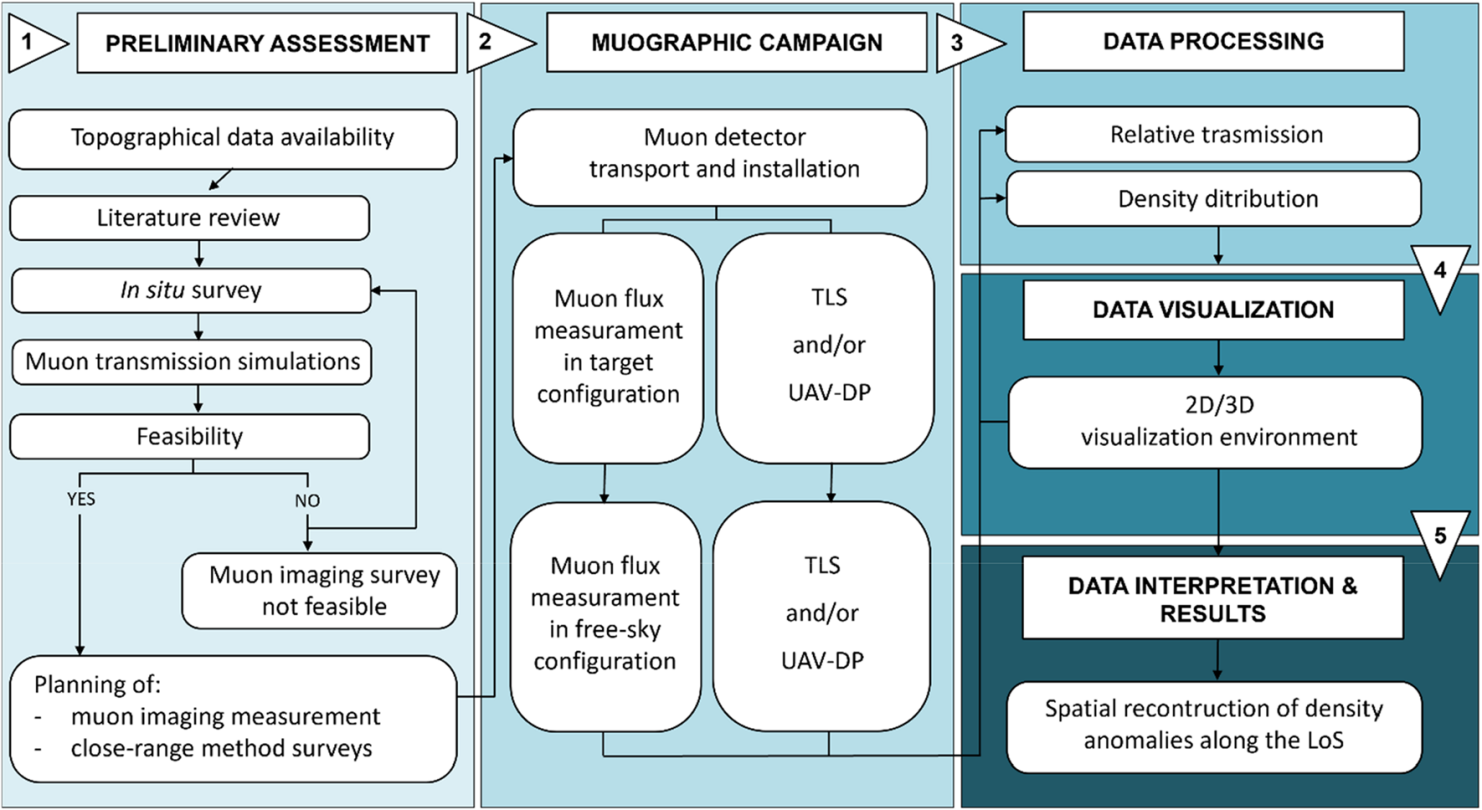
Schematic workflow followed to carry out the presented archaeological application of muography. Five main phases were defined: (1) preliminary assessment; (2) muographic campaign; (3) data processing; (4) data visualization; (5) data interpretation and results. Remote sensing methods and outputs appear throughout the workflow from phases 1 to 5.

The schematic workflow reported in Fig. 1 shows how the muon radiography technique is supported by the employment of the TLS and UAV-DP techniques and throughout the entire muographic campaign. Furthermore, the present case study, which is an archaeological application of muography (a relatively small target), stresses the importance of the RS product (point clouds and meshes) in the muon imaging analysis workflow for obtaining the length of the muon path within the studied target. Citing one of the most well-known archaeological applications of muography, Procureur et al. (2023) asserted that the existence of the north face corridor in Khufu's Pyramid was established through explicit comparison with a 3D geometric model of the pyramid. The availability of topographical data is particularly significant in the first phase of the muography survey. It is important to execute preliminary muon transmission simulations by using the geometry of the chosen measurement location. To confirm the feasibility of the muon imaging campaign in terms of the measurement time, muon rate acquisition and cavity detectability with respect to the geometric possibilities of the tracker.

## Case study

### The Palazzone necropolis (Perugia, central Italy)

**Figure 2:**
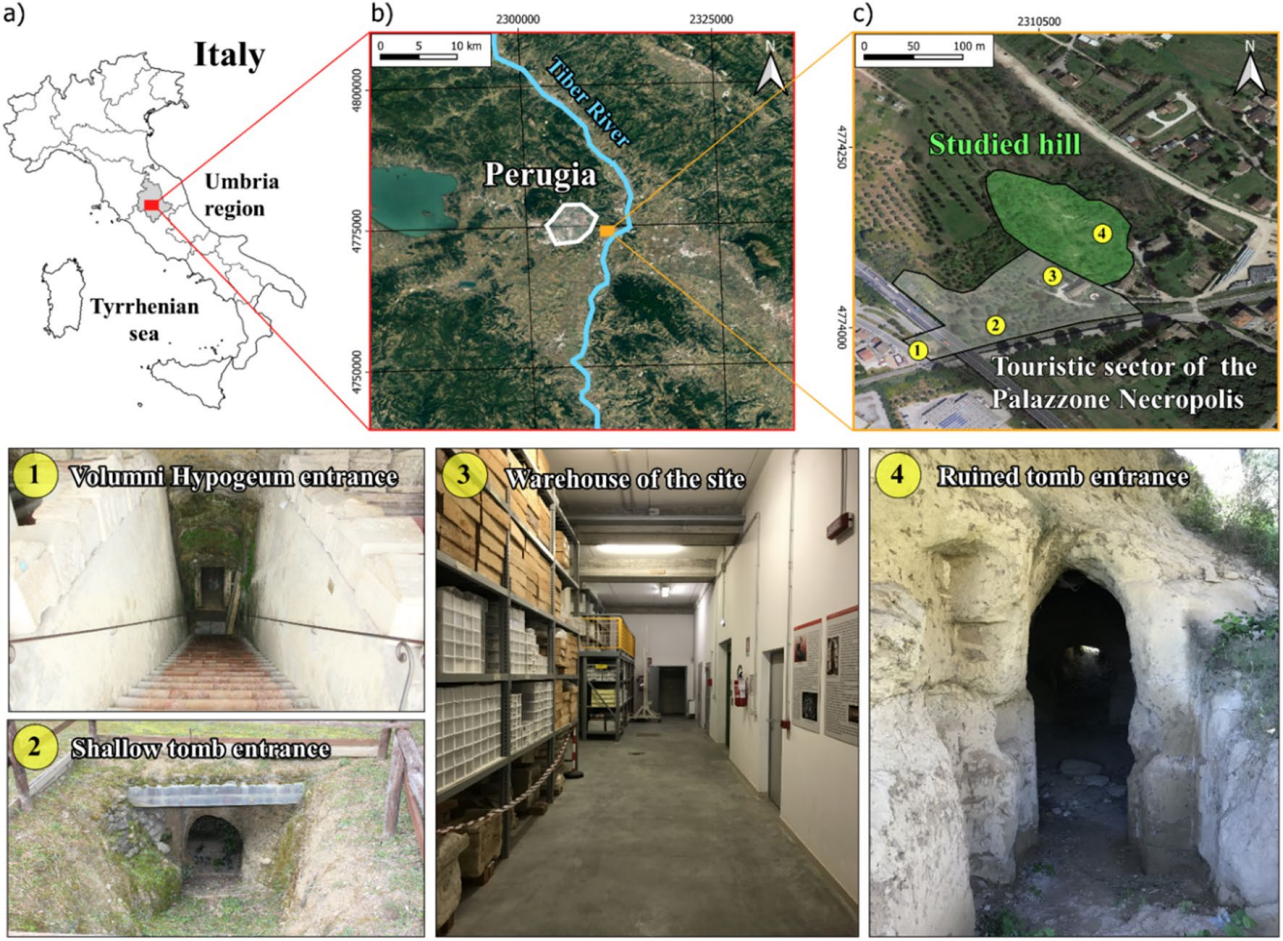
Geographic location and photos of the study area: (**a**) Italy and Umbria region; (**b**) in white, the perimeter of Perugia and, on the eastern side of the city, the area involved in this study is highlighted by the orange rectangle; (**c**) aerial view of the Palazzone necropolis, the white polygon delimits the touristic sector of the necropolis and the green one the studied area. The information reported in (**b,c**) are draped on a satellite base map uploaded on the free and open source QGIS software^50^. At the bottom of the figure, some explicative photos show some of the many underground spaces accessible at the necropolis (the position of the numbered yellow circles in (**c**). These images show the appearance of the local archaeological heritage represented by shallow-to-depth underground tombs (1, 2, 4) and the warehouse of the touristic site (3).

To demonstrate the importance of the RS methods throughout the muon imaging process, a complex real case study is presented: searching for unknown tombs in the Palazzone necropolis (Perugia, Italy; Fig. 2). The necropolis is also known as Volumni Hypogeum, from the name of the most important tomb of the necropolis (more than 200 m^3^ volume). Since the Etruscan period, the Perugia settlement (*Perusia*) has played a key role in the development of the Etruria league due to its strategic location at the top of a hill above the Tiber River (Fig. 2b).

The Etruscan settlement of *Perusia* survived the Roman period. Within the sixth and second centuries B.C., the Etruscan culture was characterized by a well-developed funerary architecture due to the belief in life after death. In this context, the necropolis of Palazzone was carved. It is characterized by the presence of almost two-hundred known hypogeum tombs (with volumes ranging from about 5 to 30 m^3^) and several potentially unknown ones^6,51,52^. These kinds of tombs were carved underground, usually at shallow depths, except in some cases that could reach a depth of up to several meters below the surface, as in the case of the Volumni hypogeum (Fig. 2c). The latter represents the most famous and well-preserved tomb of Palazzone necropolis. Generally, the entrances of tombs are carved in correspondence with slope changes and develop orthogonally to the main entrance^6,51^. The body of the tombs can be constituted by at least one single room (the smallest tombs). Overall, this necropolis is considered to be one of the most significant Etruscan funerary sites in central Italy. Furthermore, this represents an interesting case of archaeo-geosites since all the perimeter walls of the tombs show the sedimentary structures related to the paleo-depositional environments^51^.

### Geological and geomorphological setting

It was important to properly set a preliminary geological-geomorphological assessment in the study area to evaluate the feasibility of the muographic survey (step 1 in Fig. 1). Several field surveys were conducted during 2021–2023. All the collected data and insights were fundamental for the muon imaging process; for the estimation of the average density values along the LoS of the muon tracker, and for the interpretation of the obtained transmission and density maps^26,28,40,53–55^. Therefore, the first goal was to characterize the studied hill slope from geological and geomorphological points of view. This is important because there is a close correlation between the lithology, surface morphology, and potential presence of Etruscan tombs, i.e. the unknown cavities we are looking for. The tombs were carved in the most workable sand, leaving the conglomerate lenses as structural elements, that is, the roof and/or pillars.

The necropolis of Palazzone is located on the southeast side of the Perugia hill (Fig. 2b,c), which consists of Plio-Pleistocene fluvial and lacustrine deposits referred to as the paleo Tiber River basin^51,52^. In the necropolis area, these deposits are characterized by alternating sequences of sandstone and conglomerate lenses with varying flow direction dispersion ranging between SW-SSE and W-WSW. The morphological setting of the necropolis is represented by rounded hills; the steepest sectors of the slope correspond to more coherent conglomerate lenses. The geological origin of the area is related to the evolution of the northern Apennine since the Pliocene (~ 2.6 Myr ago). From the early Pleistocene, both alluvial plane and/or palustrine environments (low-energy fine-grained deposits) were overlaided/interbedded with coarser lenses of gravel and sand (high-energy deposits) in the study area. These variations in depositional environments, which are defined by varying energies, result in the actual stratigraphic succession that is plainly discernible within the accessible tombs and surficial outcrops. These outcrops allowed to carry out sedimentological survey campaigns that led to the identification of two main lithostratigraphic units: the lower Volumni Unit (Vol) and the upper Palazzone Unit (Plz)^51,52^. Within the former unit, conglomerates prevail upon the sandstone, and in the latter is the converse. Both the Vol and Plz units constitute the target-hill falling within the acceptance of the used muon detector.

## Results

**Figure 3:**
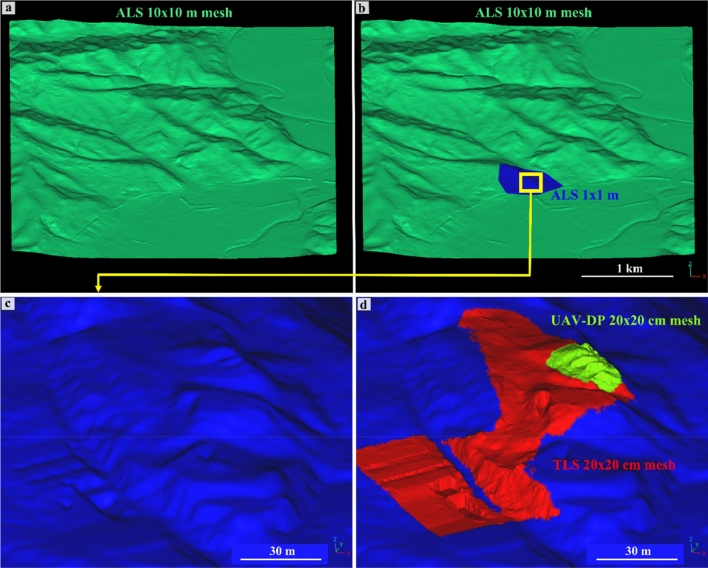
Three-dimensional processed models of the study area based on RS-acquired data. Four datasets were combined to create the most reliable and accurate three-dimensional environment to be used for muon absorption simulations within the target hill of the necropolis: ministerial ALS of 10 × 10 m^2^ and 1 × 1 m^2^ resolution, TLS of 0.02 × 0.02 m^2^ and UAV-DP of 0.2 × 0.2 m^2^. In (**a**), the mesh of the whole involved area obtained from the ALS survey, with a resolution of 10 × 10 m^2^, is shown; in (**b**), the same mesh of (**a**) was merged with the 1 × 1 m^2^ resolution on the Palazzone necropolis area; (**c**) zoom on the eastern side of the necropolis (the studied target, see Fig. 2); (**d**) ministerial data (in blue), TLS data (in red) and UAV-DP data (in green) merged together to obtain the most reliable three-dimensional reconstruction of the studied environment, comprising the most precise components from the processed point clouds. A visual comparison between (**c**) and (**d**) already offers insight into the extent of differences in the ground surface reconstruction within the two three-dimensional models.

This study confirms the importance of RS methods throughout the muography survey workflow. The availability of four topographical datasets, namely, point clouds obtained by ministerial ALS (1 × 1 m^2^ and 10 × 10 m^2^), TLS and UAV-DP techniques, made it possible to verify and quantify the potential errors arising from the employed dataset, which could affect the final muon imaging outputs (transmission and density maps). To better understand in which part of the muon imaging analysis process the RS output play a key-role we need to focus on the theory of the simulation part (data processing step in Fig. 1). In this study, the simulated transmission ${T}_{s}\left(\theta ,\varphi , \overline{\rho }\right)$ computed to achieve the muographic survey was calculated using a three-dimensional model created by merging the TLS, UAV-DP and ALS datasets (Fig. 3).

These three-dimensional data allowed to calculate, along each LoS within the MIMA detector acceptance, the rock thickness given by the distance between the center of the tracker and the terrain surface $L(\theta ,\varphi )$, assuming no cavities in addition to those surveyed e.g., the known tomb #11 (Fig. 2).

The opacity $X$ of the target with respect to the center of the MIMA detector is defined as follows:

1 $$X\left(\theta ,\varphi ,\overline{\rho }\right)={\int _{LoS}}\rho \left(\theta ,\varphi \right)dL= \overline{\rho }\left(\theta ,\varphi \right)\cdot L(\theta ,\varphi )$$

where *ρ* is the traversed matter density and $\overline{\rho }(\theta ,\varphi )$ is the average density assigned along the LoS. The Eq. (1) suggests the importance of the distance $L(\theta ,\varphi )$ in this calculation. Indeed, the opacity values ($X$) obtained depend directly on it.

The simulated transmission ${T}_{s}\left(\theta ,\varphi , \overline{\rho }\right)$ is defined in the same manner as ${T}_{m}\left(\theta ,\varphi \right)$:

2 $${T}_{m}\left(\theta ,\varphi \right)= \frac{{\phi }_{m, target}\left(\theta ,\varphi \right)}{{\phi }_{m,free-sky}\left(\theta ,\varphi \right)}$$

Using simulated muon fluxes for both the free-sky and target configurations, we define:

3 $${T}_{s}\left(\theta ,\varphi , \overline{\rho }\right)= \frac{{\phi }_{s, target}\left(\theta ,\varphi ,\overline{\rho }\right)}{{\phi }_{s,free-sky}\left(\theta ,\varphi \right)}$$

4 $${\phi }_{s, target}\left(\theta ,\varphi ,\overline{\rho }\right)={\int _{{E}_{min}(X)}^{\infty }}j\left(\theta ,\varphi ,E\right)dE$$

5 $${\phi }_{s,free-sky}\left(\theta ,\varphi \right)={\int_{{E}_{0}}^{\infty }}j\left(\theta ,\varphi ,E\right)dE$$

where $j\left(\theta ,\varphi ,E\right)$ is the parameterization of the muon flux measured by ADAMO measurements^56^, as a function of the altazimuth angles and muon energy. The value ${E}_{min}(X)$ is the lower integral limit obtained from the opacity (*X*); it assumes the minimum energy value that a muon must have to be detected by MIMA in the target configuration. It follows that ground surface errors can result in thickness errors that can systematically affect all subsequent calculations; incorrect relative transmission values ${T}_{rel}\left(\theta ,\varphi ,\overline{\rho }\right)$ could be obtained. The latter is defined as:

6 $${T}_{rel}\left(\theta ,\varphi ,\overline{\rho }\right)=\frac{{T}_{m}(\theta ,\varphi )}{{T}_{s}(\theta ,\varphi ,\overline{\rho })}$$

The Eq. (6) enables us to find anomalies, i.e. tombs or cavities, whenever an angular region within the detector acceptance cone has a value of ${T}_{rel}$ considerably different from the unity. Depending on the simulation geometry, these anomalies could be caused by density anomalies or a lack or excess of matter. Therefore, the use of a wrong three-dimensional ground model could directly affect the output density values of the muographic measurements.

Keeping this in mind, a cautelative absolute error of the surveyed ground surface at the necropolis was estimated. Each RS-obtained dataset was spatially and geometrically analyzed with the aim of reconstructing the most reliable and similar dataset to the real ground surface to run simulations. To this end, 293 ground points were acquired, with a precision of 7 mm in the horizontal direction and 14 mm in the vertical direction, using the Emlid Reach RS2 GPS in the field and used as ground truth points throughout the presented results.

### Three-dimensional data analysis

**Figure 4:**
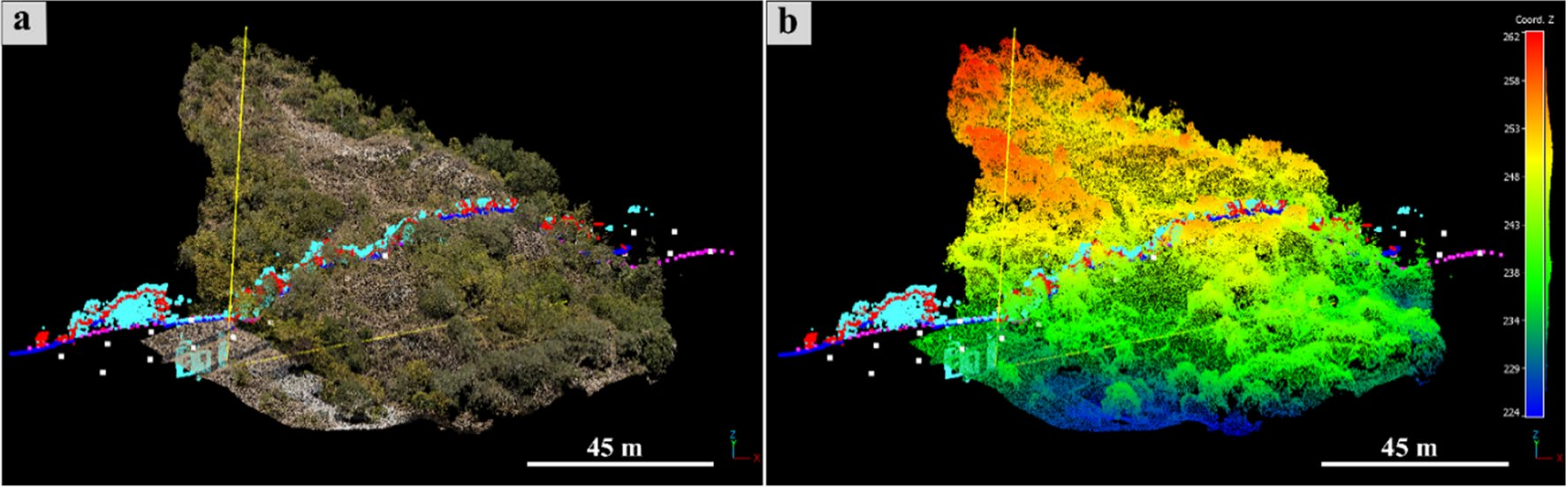
Analysis of the point cloud section draped on the UAV-DP model: (**a**) view of the analyzed data section passing through the center of the MIMA detector and “cutting” the target hill. The section consists of all the available datasets, visualized on the RGB UAV-DP point cloud; in (**b**) the same data of (**a**) are shown but with the altimetric values visualized as a color scale.

**Figure 5:**
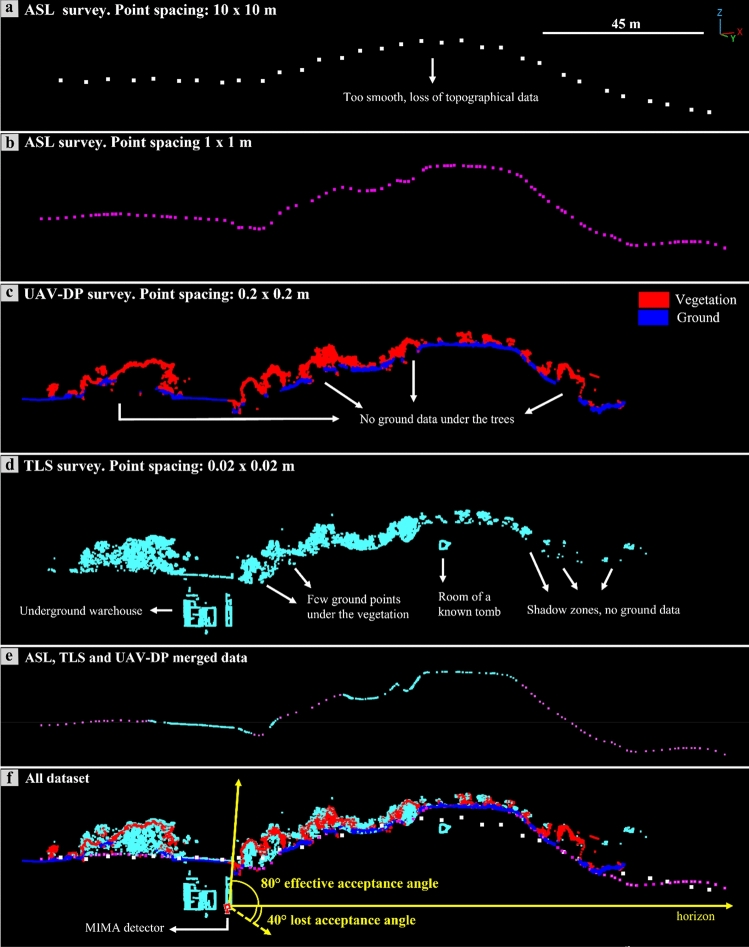
Point cloud data comparison along the section shown in Fig. 4: (**a**) section of the 10 × 10 m^2^ point cloud from ministerial ALS; (**b**) 1 × 1 m^2^ point cloud from ministerial ALS; (**c**) 0.2 × 0.2 m^2^ point cloud from the UAV-DP survey, the segmentation in ground and vegetation points shows many missing data zones under the vegetation; (**d**) 0.02 × 0.02 m^2^ point cloud from the TLS survey, few ground points are visible under the vegetation and a section of a known tomb chamber is present within the studied hill; (**e**) section of the point cloud obtained by merging the ALS,TLS and UAV-DP datasets cleaned by vegetation; (**f**) all the available datasets together with the MIMA detector location, the yellow arrows highlight the effective and lost acceptance angles.

Figure 4 and 5 show the high presence of vegetation in both the TLS- and UAV-DP-obtained point clouds of the studied scenario. After the creation and orientation of these three-dimensional dataset, the first step was to reduce the number of points required to facilitate the processing time and the subsequent cleaning process. The raw TLS dense point cloud (about half a billion points) was subsampled at 0.02 m point-to-point distance (less than 10 million points), and the UAV-DP one at 0.2 m (six hundred thousand points). The ALS point clouds of 1 × 1 m^2^ and 10 × 10 m^2^ did not need to be cleaned or scaled because they were sent to us already correct by the Ministry of Environment and Land and Sea Protection of the Umbria Region^57^. The cleaning phase was the most important and time-consuming phase, several hours were dedicated to removing unwanted manufacts, grass, bushes and trees from the TLS and UAV-DP scenes. We pointed out that the cleaning process available on the licensed software RISCAN PRO based on the filtering of returning digitized echo signals could not clearly discriminate multitargets along the TLS LoS in such a complex environment. Therefore, because of dense and ever-green vegetation, point cloud cleaning was primarily achieved manually using the CloudCompare software (CC)^58^. Approximately, 12 h were required to achieve the UAV-DP survey (flights + laboratory processing) and 24 h for the TLS survey (in situ + processing). All the oriented and cleaned point cloud data were compared along a chosen section (Figs. 4 and 5) that was traced parallel to the MIMA detector pointing direction and passing from both its center and the target hill.

In Fig. 5a–d is possible to visualize the differences and similarities among the available datasets along the section shown in Fig. 4.

It is well observable that there is a loss of spatial information for the dataset with higher point-to-point distances (see Fig. 5a with respect to Fig. 5b–d) and that the UAV-DP and TLS surveys (Fig. 5c, d) both suffer from shadow zones caused by the presence of vegetation. Nevertheless, TLS data provide more three-dimensional information about underground spaces; otherwise, it is impossible to achieve the latter using UAV-DP and ALS methods. The underground geometry obtained from the TLS data was fundamental for inferring the real position of the MIMA detector center underground with respect to the ground surface, allowing the calculation of $L(\theta ,\varphi )$. By leveraging the TLS point cloud data obtained underground, which includes the tracker that was acquiring, we were able to calculate the orientation of several best fitting planes and lines corresponding to the faces and edges of the cubic MIMA tracker, to infer the location of the center and the pointing direction (in addition to the angle returned by the analog clinometer shown in section "Material and methods"). This process facilitates the determination of the tracker center, which is crucial for accurate simulations. The aim was to minimize potential misalignments between the tracker's position in the simulations and the actual location in the target configuration (real measurements obtained underground). Such misalignments can introduce bias and errors in the final relative transmission and density maps. Figure 5e suggests that the best way to recreate the real ground surface of the studied hill is to merge different datasets in a single point cloud (see Fig. 3d). Figure 5f allows the visualization of the differences among all the RS-obtained point clouds and to understand how easy it is to introduce errors in the detector-to-surface distance considering the wrong ground dataset. The red square in Fig. 5f represents the MIMA detector inside the warehouse pointing toward the target hill, and the yellow lines show a two-dimensional view of the acceptance cone of the tracker. With respect to the horizon, we lost 40° of acceptance and used 80° as the effective acceptance angle (zenith angle $\theta$).

**Figure 6:**
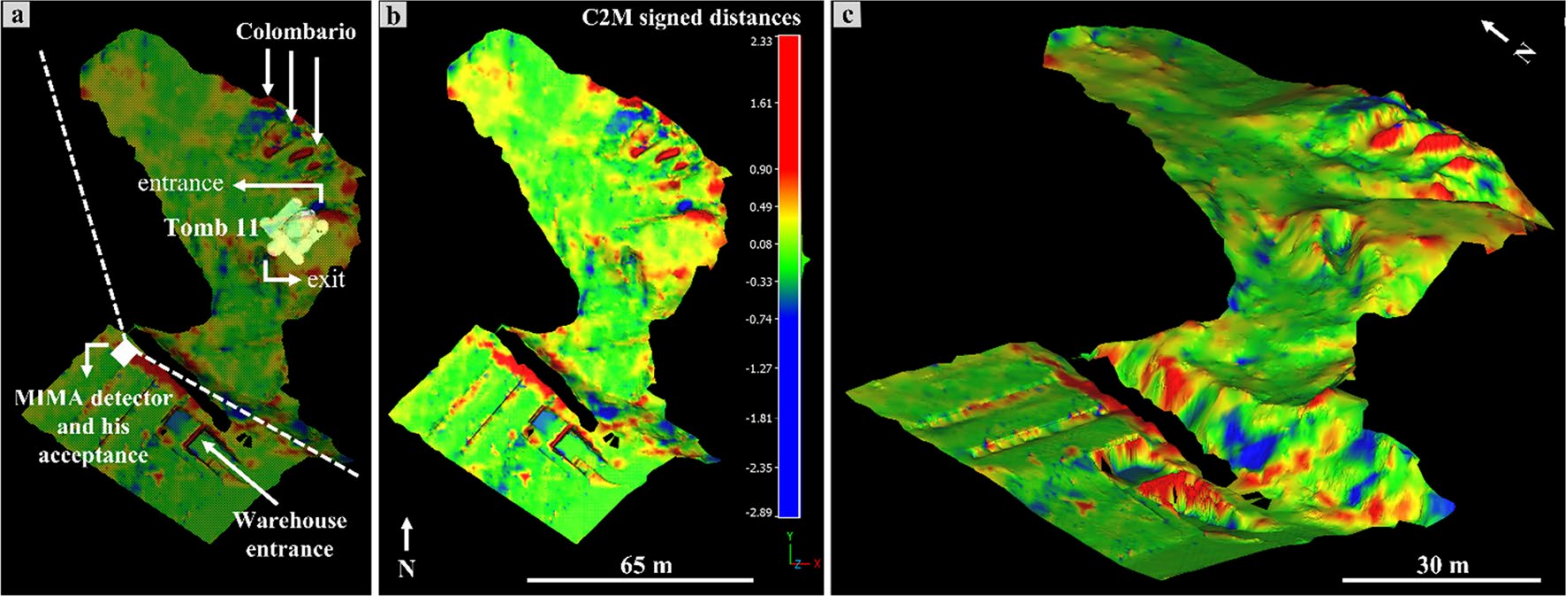
Elevation differences along the z-axis draped on the best three-dimensional model of the studied area: (**a**) Cloud-to-Mesh (C2M) signed distance calculated between the 1 × 1 m^2^ ministerial ALS mesh and the point cloud obtained by merging UAV-DP and TLS point clouds. Underground tomb #11 is shown, and the white dotted lines represent a two-dimensional view of the MIMA detector acceptance cone. In (**b**), the same orthogonal view of (**a**) without transparency, arrows, lettering and, in (**c**), the using a different view angle.

Using the cloud-to-mesh (C2M) tool available on CC^58^, we preliminarily calculated the elevation differences along the Z-axis between the mesh of ALS 1 × 1 m^2^ and the merged TLS-UAV point cloud in the same area falling within the MIMA detector acceptance (Fig. 6).

The highest positive differences along the z-axis, that is, points of the TLS-UAV point cloud that are located higher with respect to the ALS 1 × 1 m^2^ mesh, reach a maximum value of approximately 2.3 m and the lowest negative ones, i.e. the points of the TLS-UAV point cloud that are located lower with respect to the ALS 1 × 1 m^2^ mesh, reach a minimum value of -2.9 m (Fig. 6). The most troubled areas, characterized by the higher negative and maximum deviation, are the top of the warehouse, the entrance of tomb #11, and the Colombario sector (red and blue areas; Fig. 6). However, the warehouse areas falling behind the MIMA acceptance cone (Fig. 6a), were not considered in the muon imaging analysis whereas the colombario ones, located at the center of the acceptance cone, has been included in the thickness calculation and analysis. The elevation differences calculated along the z-axis allowed us to focus our attention on the zones where potential errors could arise^6,13,15,59^ and on these suggestions, trying to precisely quantify these elevation differences with respect to the true ground surface using the available GPS-acquired ground surface points. At this stage of the analysis, it is clear that the same studied target can restitute different density maps after the muon imaging survey, depending on the availability of three-dimensional data.

**Figure 7:**
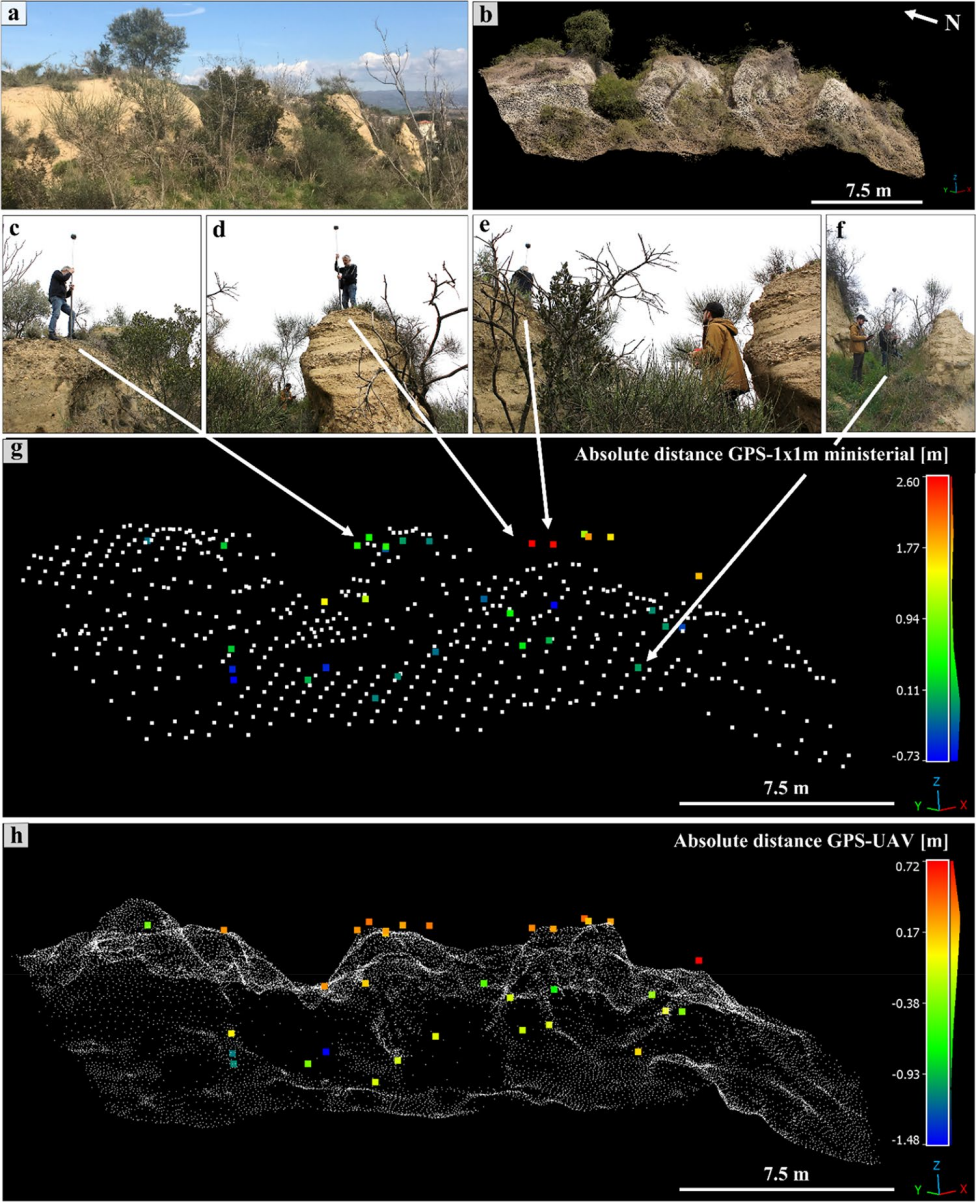
Three-dimensional analysis of the Colombario area: in (**a,b**), the RGB image and the UAV-DP obtained point cloud of the scene, relatively. (**c–f**) Photos of the GPS in situ survey to obtain the ground truth. In (**g,h**) the C2C absolute distance values computed on CC between the GPS-acquired points and the ministerial 1 × 1 m^2^ ALS and UAV-DP datasets, respectively.

**Figure 8:**
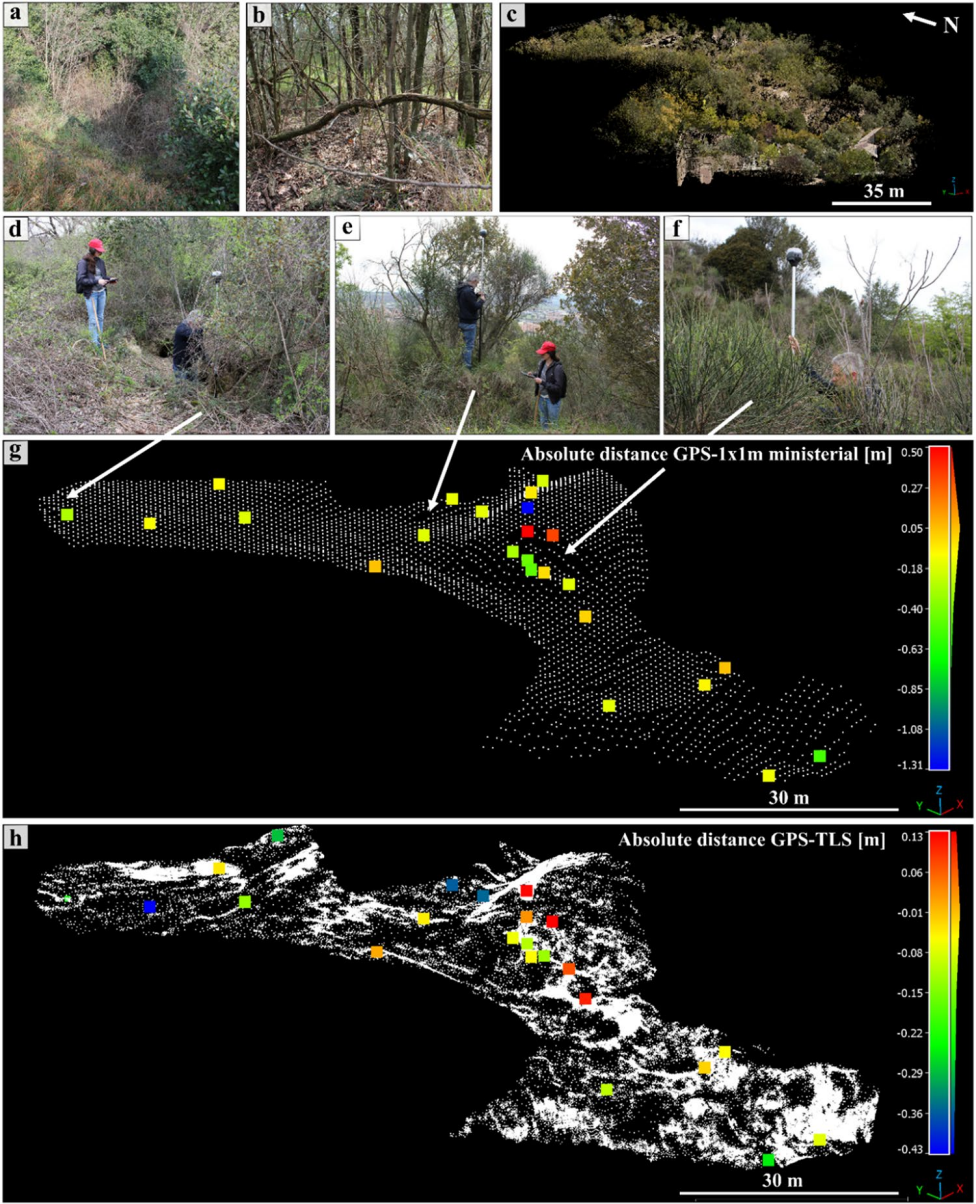
Three-dimensional analysis of the hill without the Colombario area: in (**a,b**) photos of the dense vegetation that characterizes the ground hill. In (**c**) the TLS obtained point cloud of the scene. (**d–f**) Photos of the GPS in situ survey to obtain the ground truth. In (**g,h**) the C2C absolute distance values computed on CC between the GPS-acquired points and the ministerial 1 × 1 m^2^ ALS and TLS datasets, respectively.

To obtain the most affordable results, we carried out a detailed GPS survey; 293 points were measured with centimeter precision over the entire study area and used as the ground truth references, with respect to the TLS, UAV, and ALS datasets, to quantify punctual elevation differences. The values obtained from the C2M analysis between the ALS and TLS-UAV point clouds (Fig. 6) suggested that the target hill could be divided into two areas: the Colombario area (Fig. 7) and the remaining part of the hill (Fig. 8). For both chosen areas, two three-dimensional datasets were available: 1 × 1 m^2^ ministerial ALS and UAV-DP for Colombario, and 1 × 1 m^2^ ministerial ALS and TLS for the other. The gaussian fitting of the C2C-obtained height difference values returned a mean difference value *m*_*d*_ = 0.38 m with a sigma *σ* = 0.90 for the GPS-ALS comparison (Fig. 7g), and *m*_*d*_ = -0.07 m and a *σ* = 0.48 for the GPS-UAV comparison at the Colombario area (Fig. 7h). The same approach applied for the remaining area returned an *m*_*d*_ = -0.17 m and a *σ* = 0.32 for the GPS-ALS comparison (Fig. 8g) and a *m*_*d*_ = -0.11 m and a *σ* = 0.15 for the GPS-TLS comparison (Fig. 8h). These results highlight that the most accurate datasets to be selected for the muography simulation processes were the UAV-DP point cloud for the Colombario area and the TLS for the remaining hill because of their low elevation differences with respect to the ground truth, that is GPS-acquired points.

### Depth map analysis

To quantify the influence of ground surface elevation differences on muon imaging, the detector-to-surface distance was calculated for each LoS of the MIMA detector for each dataset. The angular map representing the obtained thickness value (*L*) for each $(\theta ,\varphi )$ is called depth map. Every bin of the depth map assumes the metric value of the crossed matter thickness between the center of the muon tracker and the ground surface.

**Figure 9:**
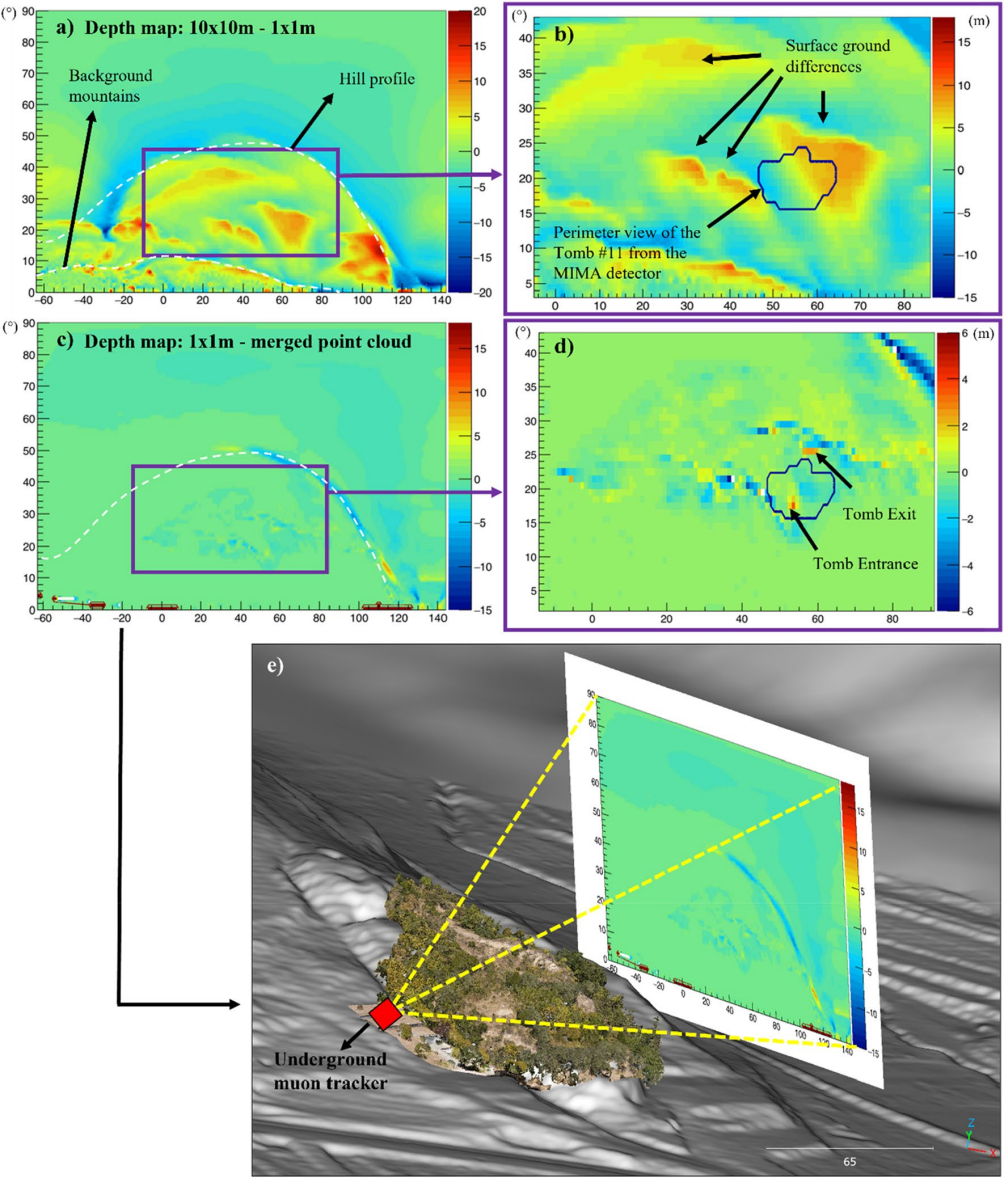
Depth maps differences calculated along the LoS of the MIMA detector that was placed in the underground warehouse of the necropolis: (**a**) depth map obtained by comparing the 1 × 1 m^2^ with the 10 × 10 m ALS-obtained ministerial surfaces, on the X–Y axis the degree from the center of the tracker, the color scale shows the depth differences in meters, and the dotted white lines highlight the perimeter of the studied hill and that of the higher mountains behind. The differences between these two datasets can reach up to ± 20 m; (**b**) zoom of (**a**) showing in blue the perimeter of the known tomb #11 located inside the hill surveyed during the TLS campaign, (**c**) depth map obtained by comparing the 1 × 1 m^2^ ministerial surface with the ground surface obtained merging all the available data (UAV-DP, TLS and the 1 × 1 m^2^ ministerial; see Fig. 3d). In (**d**) the zoom of (**c**) allows to understand the surficial differences among the compared datasets. In (**e**) a three-dimensional view of the depth map is shown in (**c**) to facilitate the understanding of this kind of map.

To corroborate the importance of choosing the right three-dimensional dataset, three depth maps were created, i.e. one map for each dataset (TLS-UAV, ministerial 1 × 1 m^2^ and 10 × 10 m^2^ ALS, TLS-UAV), using a custom algorithm that works with meshes (Fig. 9). Next, we computed the thickness differences between the three created depth maps: 10 × 10 m^2^ ALS less 1 × 1 m^2^ ALS (Fig. 9a) and 1 × 1 m^2^ ALS less TLS-UAV (Fig. 9c). The obtained results show an excess of thickness and defects ranging up to ± 20 m along some unfortunate $\left(\theta ,\varphi \right)$ areas (red and blue bins in Fig. 9). The analysis of the depth maps differences allowed to visualize, identify and isolate the potential affected areas, those are primarily represented by the hill slope surface parallel to the LoS of the detector (see Fig. 9e) along which ± 0.50 m could become up to ± 20 m of non-real deficit/excess of matter thickness. Considering that the maximum dimension of the tombs can reach approximately 5–10 m, it is easy to see that the worst topographical dataset to use is the ministerial 10 × 10 m^2^ ALS and the best one is obtained by merging the TLS and UAV-DP data. Several details, such as the hole of the entrance/exit of tomb 11 and other cavity/pit/hollows and recent earth deposits throughout the area, are lost using the ministerial 1 × 1 m^2^ ALS dataset but not using the TLS-UAV merged dataset (Fig. 9d). This key passage allows us to quantify and map all areas affected by non-negligible errors within the MIMA detector acceptance. It is suggested to exclude these areas during muon imaging analysis or to treat these carefully taking into account the errors on *L*. These inaccuracies in thickness estimation can propagate considerably over the estimate of *T*_*s*_, and therefore *T*_*rel*_, and can be a source of density artifacts in muon imaging density maps, i.e. they can generate non-real anomalies of density, namely tombs or cavities in the target hill that do not exist. Especially considering that in the archaeological application we are presenting, the dimensions of the tombs we are searching for are comparable to the possible inaccuracies affecting the shallow strata on the hill slopes.

The values of *L* $\left(\theta ,\varphi \right)$ shown in the depth maps are those that could be inserted in Eq. 1 to carry on the muography analysis. Thus, the availability of more accurate RS survey data, with respect to the ministerial data, allowed us to make the muographic campaign at the Palazzone necropolis feasible and reliable.

## Discussion

To date, several non-invasive geophysical methodologies have been applied simultaneously to solve archaeological issues and questions. This multidisciplinary approach is commonly used in archaeological heritage research^60^. The use of muography techniques coupled with other well-established geophysical techniques, such as ground penetrating radar (GPR) and electrical resistivity tomography (ERT), is becoming a real possibility as a complementary method for geophysical non-invasive surveys^37^. Several authors have confirmed the maturity of muon imaging techniques, i.e. muon tomography and muon radiography, to be launched as commercial techniques^35,48,61,62^. Recent studies have confirmed the potential of muography measurements applied to synthetic and/or real case studies, suggesting a wide range of possible applications^27,31,34,36,37,40,41,55^. Nevertheless, no author has gone deeper into parallel multidisciplinary activities such as the use of RS methods as part of the muon radiography process. Only a few studies have highlighted the constraints of RS methods within the muon imaging workflow. In fact, the creation of a three-dimensional environment is a fundamental step for the achievement of a real muon radiography application when simulations are needed to assign directional relative transmission values and then convert them into directional average density values^26,41,55,63^.

In the literature, only brief references and descriptions are given on the techniques used to obtain the three-dimensional environment and to infer the coordinates of the center of the used detector, which are crucial information for carrying out the muon flux simulation. This point has rarely been discussed in the context of muographic measures, and little is known about the potential errors introduced in muon imaging outputs. A detailed case study that deals with this type of observation was reported by Procureur et al. (2023). On the contrary, much attention has been usually given to the physics-related issues of muons, in particular to the evaluation and quantification of the multiple Coulomb scattering phenomenon in muon transmission imaging of large targets. Multiple scattering consists of muon flux deviation within the crossed matter, depending on the thickness and composition of the target. It makes sense to raise such observations because the muons detected after scattering contribute to the increase in the background noise, and this influences the accuracy of the final muography-obtained image, mainly along the contacts between different densities^55,64–66^. Attention is also given to the evaluation of errors arising from the employed muon flux generators (the muon flux model used) and the following data processing.

The aim of the present work is to highlight how the sources and propagation of potential errors in the muon imaging process depend on both the muon behavior physics within the matter and the accuracy of the created environment, i.e. the three-dimensional model of the target ground, particularly for archaeological applications (smaller targets).

The errors to be examined vary from case study to case study and mostly depend on the objective of the muography campaign. For instance, in preliminary archaeological investigations (small targets, not mountains or volcanoes), it is possible to use transmission-based muography to evaluate potential excavation zones, that is, areas of lower density, without worrying about their precise shape and edges (i.e. not considering multiple scattering in the transmission and density maps) and tolerating up to metric errors along the contacts of the identified density anomalies. However, in the field of archaeological research, accurate information about a target may be required, as in the case of muon imaging applied to pyramids^38,39,41^. For example, in Procureur et al. (2023), the characterization of a corridor-shaped structure in Khufu’s Pyramid is presented and, in this case, due to the importance of the studied cultural heritage, the need to consider all the potential errors resulting from the multiple Coulomb scattering, the used muon flux model and those due to the used RS method to create the geometry of the pyramid (external surface and internal voids/chambers/tunnels), in order to reconstruct, as accurately as possible, the location of the muon detector and the shape of the unknown chamber and correctly locate it in the space for further study (direct survey), it is justified. Therefore, operators searching for small targets of archaeological interest, namely cavities of meters, at a distance of meters/tens of meters from the detector (unknown tombs, rooms, and/or passages) could decide whether or not to consider as many potential errors as possible, depending on the significance of the studied target and the survey's purpose, i.e. preliminary or deeper insights.

The central idea of this reasoning is to recognize that the errors resulting from the available DEM or DTM, i.e. the used three-dimensional topography, must sometimes be considered at the same level as the other errors resulting from the instrumentation, multiple scattering phenomena and the used muon flux model. The accuracy of the topography directly depends on the in situ survey issues, i.e. presence of vegetation and shadow zones, which are not easy to solve. Therefore, it is essential to conduct a preliminary assessment survey to immediately evaluate the feasibility of the muographic campaign with respect to the available topographical data (ministerial ALS or DTM) and the possibility of performing TLS and/or UAV-DP surveys. For the Palazzone necropolis, the authors pointed out that by combining three RS survey techniques, it was possible to create a reliable three-dimensional environment, reaching a cautelative ground surface uncertainty of maximum ± 0.5 m on the whole surveyed hill. The topographical errors range between ± 0.50 m, on the z-axis for mesh triangles with an orientation orthogonal to the LoS of the detector and up to ± 15 m for mesh triangles oriented parallel (the slope surface dipping to the center of the detector). The importance of these errors depends on the target dimensions; for example, if the target is a volcano or deep mine, topographical errors will probably become negligible with respect to other sources of errors (detector-specific noise, contamination of low-momentum particles, systematic biases due to calibration errors in detection efficiency and errors in secondary cosmic ray muon fluxes). In archaeological applications such as the one presented here, the target hill thickness ranges from a minimum value of 60 m to a maximum of 120 m, and the tombs we are looking for range from 2 to 10 m in height and length. For the LoS parallel to the slope surface dip direction/dip, we reached (on average) a density error of up ± 0.4 g/cm^3^. Regarding the other sources of errors due to the geometry and location of the detector and the data analysis process (in particular, the transmission-density conversion procedure involving simulation), we reached (statistical error) ± 0.1 g/cm^3^. This achievement allowed us to not consider all the potential LoS areas affected by topographical elevation errors that could lead to non-real thickness values (see depth maps in Fig. 9).

## Material and methods

A key-role within the muon imaging analysis workflow (Fig. 1) is played by the availability of a three-dimensional environment of the studied target^37,40,41,55,67^. The latter can be represented by a real three-dimensional dataset of the target or by a synthetic reconstruction of the same, i.e. a geometric model created using Geant4 or some of the other available three-dimensional modeling software (CAD, Blender 3D, SketchUp, Cinema 4D, etc.). The virtual environment must be as robust as possible because of the affordability of muography survey outputs (transmission and density maps) strictly lie on it. Several studies have confirmed this statement^34,36,37,40,63^. Indeed, to preliminarily characterize the whole study area at a regional scale, and to perform preventive feasibility muon transmission simulations, a detailed digital elevation model (DTM) is warranted. Following the proposed workflow (Fig. 1), the starting point for the preliminary assessment (phase 1) was to find a topographical dataset to start and as many insights from literature, or online portals, as possible, about the study area. Therefore, a DTM with a ground resolution of 1 m was obtained for the study area upon request from the Ministry of Environment and Land and Sea Protection of the Umbria Region^57^. This dataset was obtained from an ALS campaign acquired under the “Extraordinary Plan of Environmental Remote Sensing,” concerning the survey of the river rods of I and II orders, with respect to the hierarchical order reported in the Military Geographic Institute (IGM) catalogue of rivers. This elevation dataset allowed us to establish a starting point for planning the study and analyzing the feasibility of a muographic survey for detecting unknown tombs in this environment. Subsequently, it was possible to spatially locate the archaeological site of the Palazzone necropolis within the Umbria region (Italy) and start the preliminary assessment (Phase 1 in Fig. 1). Once the study area has been identified and the feasibility of muon radiography measurement verified, an in situ survey was carried out to check the operating space for the muon detector underground, energy supply, ethernet connection and if it was possible to plan and carry out the close-range method surveys together with the muography one.

Generally, different RS survey methods can be used to obtain a three-dimensional model of the studied target. As stated before, three well-established close-range methods were utilized for this study: (1) digital terrain model (DTM) 1 × 1 m^2^ and 10 × 10 m^2^ obtained from the ALS ministerial data; (2) in situ terrestrial laser scanner survey (TLS); and 3) in situ unmanned aerial vehicle digital photogrammetry (UAV-DP). Each technique is characterized by a different resolution: 1–10 m for airborne data, decimetric for UAV-DP data, and centrimetric/millimetric for TLS data. It follows that during the managing and analysis phases of the three different three-dimensional database the machine-time computing varied significantly from seconds to hours depending on the dataset. The technical information of the instruments used in this study are reported in Table 1. Furthermore, an in situ GPS survey, using an Emlid Reach RS2 GPS (Table 1), was conducted in the study area to quantify the accuracy of each RS dataset.

**Table 1:** Technical information of the used devices: DJI Mavic Air 2 (UAV-DP), RIEGL VZ-1000 (TLS), MIMA detector (TM) and Emlid Reach RS2 (GPS).

Devices	Sensor accuracy (’’)	Field of view (°)	Pixels (M)	Lens (mm)	Weight (kg)
DJI Mavic Air 2	0.57	84	12—48	24	0.57

	Accuracy (mm)	Divergence (mrad)	Resolution (arcsec)	Angular step (°)	Weight (kg)
RIEGL VZ-1000	5	0.3	1.8	0.0024–0.288	9.8

	Angular resolution (mrad)	Acceptance cone (°)	Consumption (W)	Dimension (cm^3^)	Weight (kg)
MIMA detector	7	120	30	50 × 50 × 50	60

	H precision RTK (mm)	V precision RTK (mm)	Convergence time (s)	Dimension (mm)	Weight (g)
Emlid Reach RS2	7	14	~ 5	126 × 126 × 142	950

### TLS survey

**Figure 10:**
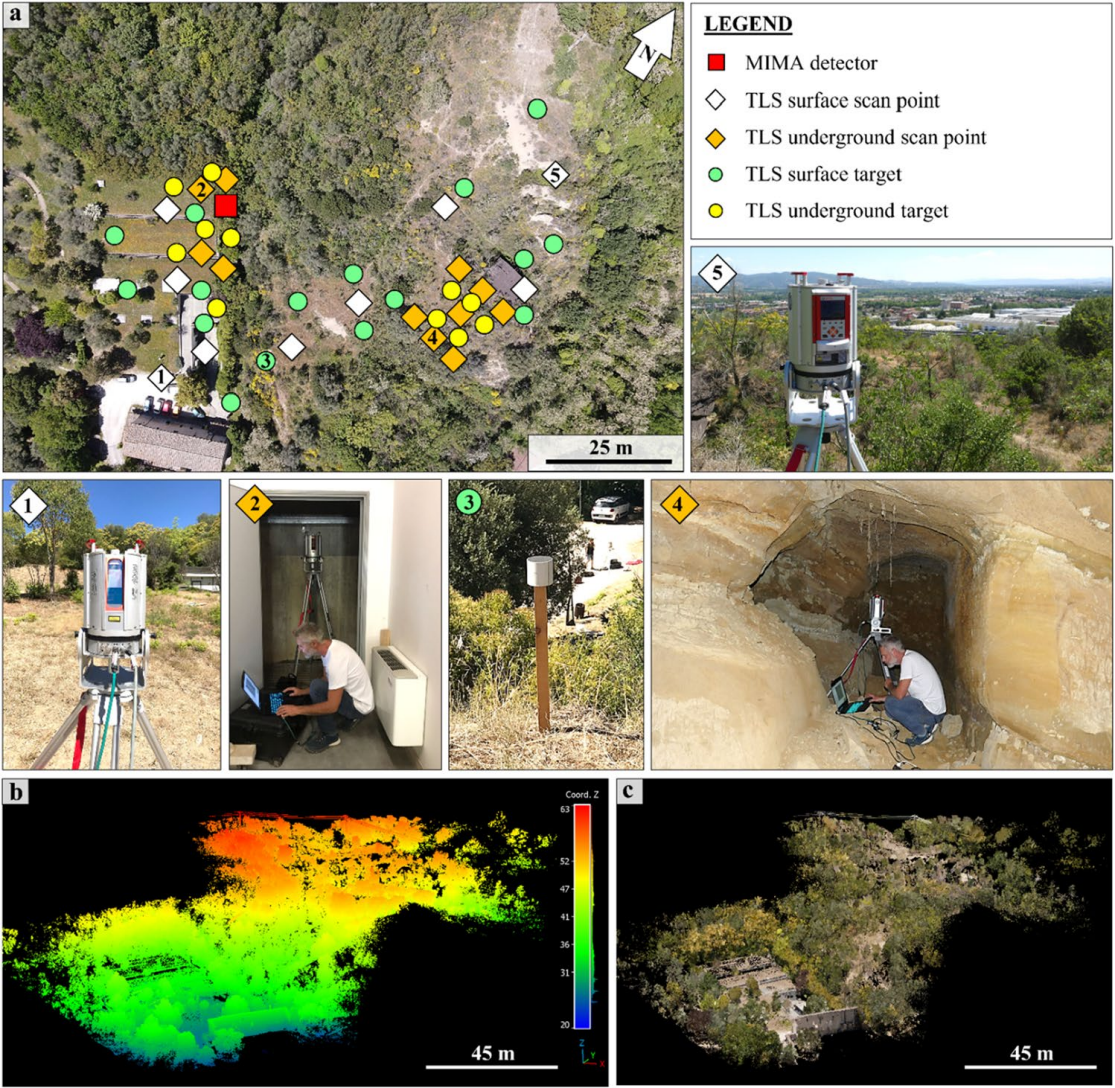
The TLS survey campaign was carried out using a RIEGL VZ-1000: (**a**) aerial photo of the study area taken from the UAV survey, in which every underground/surficial scan point and target is highlighted and some photos relative to some of these points; photos 1 and 5 show the TLS measuring in two different locations, photos 2 and 4 show the underground measurement point in the warehouse and inside a known ruined tomb, photo 3 shows one of the thirty used reflectors. In (**b**) the TLS-obtained point cloud (more than 150 million points) with elevation values. The same point cloud is shown in (**c**) with the RGB values inferred from the UAV-DP data.

A TLS campaign was conducted to establish an accurate and reliable three-dimensional survey of the studied target, namely the eastern portion of the Palazzone necropolis. The major purpose was to accurately arrange the laser scanner survey in terms of the quantity and positions of scanning locations, relative to the subsurface measuring point of the MIMA detector (muon tracker). Owing to the complicated geomorphology of the location and the abundant vegetation (grass, shrubs and trees) in both summer and winter, great care has been taken to minimize potential georeferencing problems and shadow zones. This type of difficulty was resolved by combining the data provided from the two available close-range survey methods (UAV-DP and ALS) with an in situ GPS campaign that helps fill in the missing data zones. The employed TLS system was a RIEGL VZ-1000V-line^®^ 3D Terrestrial Laser Scanner (Fig. 10), which was used without mounting the optional optical camera (no RGB data available). This high-speed TLS operates with a narrow infrared laser beam (Class 1) and fast-scanning mechanism (technical information in Table 1). The speed of acquisition reaches up to 122.000 points per second. For a target located at a distance of 100 m from the measurement point, the instrument accuracy and precision, in a standard deviation, are 8 mm and 5 mm, respectively^68^. The output is represented by a point cloud in which the X, Y, and Z values of each point are known with respect to the TLS scan position, and a scalar field of laser-returning intensity is given.

Twenty scan points were required to complete the planned campaign on the studied area. Among them, eleven were underground scan points inside the warehouse (where the MIMA detector was placed) and the ruined tomb (also known as tomb #11), and the other nine surface scan points were scattered around the entire area, from the roof of the warehouse to the eastern side of the hill (Fig. 10). The chosen angular resolution for the TLS was 0.035° for both the X (horizontal) and Y (vertical) axes, and an average of approximately 25 million points were acquired for each of the twenty 360° scan points.

To properly georeference and merge the obtained twenty TLS point clouds (a cloud for each scanning point), sixteen surface targets of known positions were used (reflectors in Fig. 10) together with eleven underground targets. GPS positions were acquired only for the surficial reflectors and not for the underground reflectors because of the absence of satellite signals below the surface. Underground reflectors were used only as reference points for the underground scans (orange dots in Fig. 10a).

The TLS survey was successfully achieved after one day of in situ measurement and two days of data processing and orientation. The survey was carried out at the same time as the muographic measurement to determine the correct position and pointing direction of the MIMA detector measuring underground relative to the entire three-dimensional environment. A single point cloud of 488,697,545 points was created and then filtered and cleaned of unwanted objects (vegetation and manufacts). The latter goals were achieved by applying point cloud segmentation both manually and using semi-automatic algorithms available on the RISCAN PRO licensed software and for free on the CC software^58^ using the CANUPO suite^59,69^. The authors pointed out how difficult it was to obtain a clean and correct ground surface of the studied hill in such a vegetated area characterized by a complex surface morphology, that is, several potential shadow zones due to rapid changes in slope angles caused by human exploitation of the site during the last two thousand years. These difficulties have also been highlighted by other authors^6^.

### UAV-DP survey

**Figure 11:**
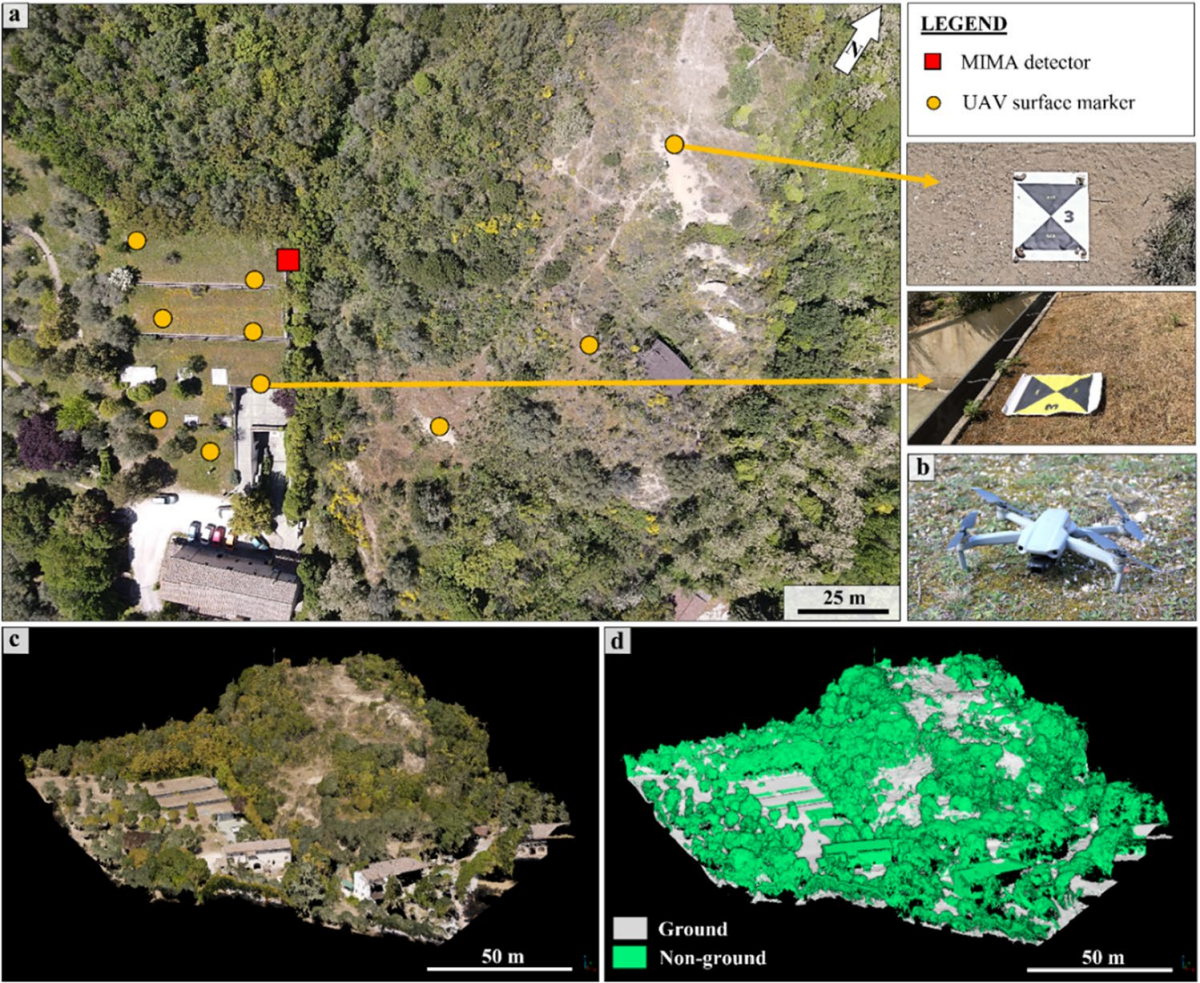
Point cloud data from the UAV-DP survey: (**a**) aerial photo of the study area taken from the UAV survey, ten surface markers were used to correctly georeference the created three-dimensional model; (**b**) the Employed UAV, a DJI Mavic Air 2 (see Table 1 for technical information); (**c**) the RGB dense point cloud of the study area, about 55 million of points; (**d**) visualization of the classified UAV-DP-obtained point cloud, two classes were identified: ground and non-ground points (vegetation and manufacts).

Currently, the use of unmanned aerial vehicles (UAV) to perform digital photogrammetry (DP) has become a consolidated approach to rapidly obtain a three-dimensional environment of the studied site^3,11,70^. The UAV-DP process requires a UAV equipped with an optical camera and several terrestrial markers that work as ground control points (GCPs), that is, the points needed to carry out the georeferencing process of the UAV-DP-obtained model and to quantify the related deformation errors (the UAV and markers are shown in Fig. 11). UAV flights can be carried out in both manual mode and automatic flight plan. The former is suggested for complex situations and relies on operator flight skills; the latter is suggested for planar scenes and/or when an affordable DTM of the area is available and could be used to maintain the same camera-to-ground distance throughout the shooting, i.e. maintain a constant ground sample distance (GSD). For the presented case study, a DJI Mavic Air 2 (Fig. 11b) was employed, and its technical information are reported in Table 1.

The UAV was manually piloted over the entire study area twice: during the preliminary survey and during the muon tracker acquisition period (phases 1–2 of the workflow shown in Fig. 1).

A GSD of ~ 3 cm was obtained. More than 500 images were captured during each flight, and all the acquired data were processed using two software packages: Pix4D^71^ and Agisoft Metashape^72^. Using the structure from motion algorithm (SfM) of the employed software, it was possible to create a three-dimensional model of the hills from the acquired RGB images. The best model obtained was chosen and georeferenced using GCPs, as shown in Fig. 11. The position of each marker was measured with centimeter accuracy using the Emlid Reach RS2 GPS in the same way as the TLS targets.

In addition to the few markers (GCPs), 293 ground points were acquired using GPS throughout the area (the studied hill) with the aim of obtaining an accurate ground point database for use as a reference in calculating the potential errors affecting the RS-obtained point clouds.

### Muon imaging with the MIMA detector

Muography is a non-destructive surveying method based on the measurement of natural atmospheric muon flux. It allows to obtain images of the inner part of the studied object, i.e. the target. The muon imaging process is comparable to that of X-ray radiography. Both methods rely on the attenuation of particles or radiation within the studied material to derive density values along various lines of sight (LoS), within the acceptance cone of the instrument.

**Figure 12:**
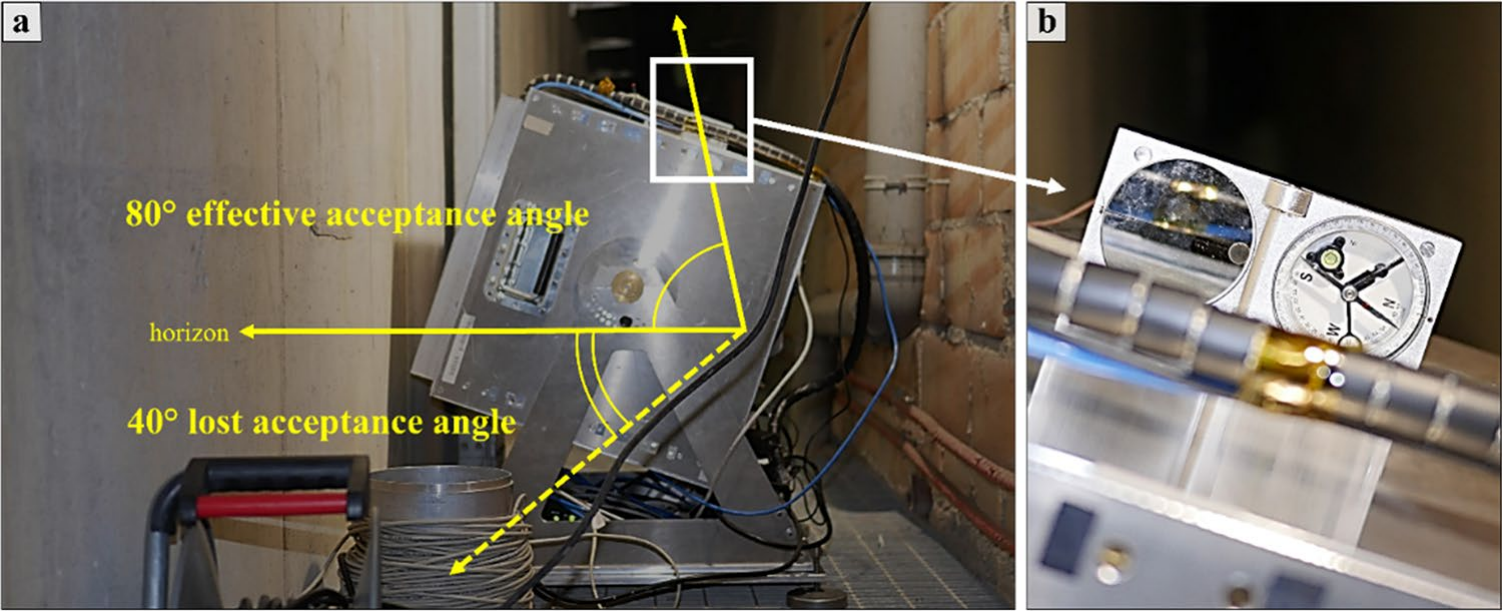
In (**a**), the MIMA detector measuring the muon flux underground in a side corridor of the warehouse of the Palazzone necropolis. In (**b**), zoom on the upper clinometer shows a pointing direction of (20 ± 1)° with respect to the horizon. Some technical information about the MIMA detector are reported in Table 1.

For this study, the Muon Imaging for Mining and Archaeology (MIMA) detector was employed to highlight potential zones of archaeological interest (unknown Etruscan tombs) in the part of the necropolis not accessible to tourists (Fig. 2c and Fig. 12).

The MIMA detector (or tracker) was developed by the National Institute for Nuclear Physics (INFN) unit of Florence and the Department of Physics and Astronomy of Florence (Fig. 1b)^73^, and the DAQ and front-end electronics of the tracker were assembled based on the knowledge and expertise acquired in the MURAVES experiment (MUon RAdiography of VESuvius)^74^. The MIMA tracker is a rugged cubic muon tracker prototype (0.5 × 0.5 × 0.5 m^3^), it is composed of three modules of coupled orthogonal tracking planes, each composed of 21 plastic scintillator bars. It can reconstruct tracks of hitting muons that fall within the acceptance cone (± 60°), resulting in a conic mapping volume. It has an active surface area of 0.16 m^2^ and requires at least one million events (tracked muon passages) in the target configuration to obtain statistically reliable data regarding the surveyed target. In this study, during the muon transmission simulations of phase 1 (Fig. 1), we pointed out that the expected acquisition time to measure one million trigger events was approximately 60 days for a pointing direction of 20° from the horizon.

Some technical information of the MIMA tracker are reported in Table 1 (for more detailed information about the hardware, the software and the followed muon imaging process, see^40,73,75,76^). In the following lines we briefly describe the muon imaging workflow.

Generally, the process of obtaining a two-dimensional density map of the studied target using muography can be summarized in three main steps^40^:1) target configuration measurement, 2) free-sky configuration measurement, and 3) comparison between the measured and simulated transmissions. During the first step, muon flux measurement is performed by pointing the detector to the studied target (${\phi }_{m,target}\left(\theta ,\varphi \right)$) and obtaining the number of crossing-target muons along each direction ${N}_{m}\left(\theta ,\varphi \right)$ within the detector acceptance. The second step consists of a free-sky muon flux measurement ${\phi }_{m,free-sky}\left(\theta ,\varphi \right)$, which has the same measurement setting as step one but without any target/objects in front of the tracker. The ratio between the measured muon flux in the target configuration and free-sky configuration is the measured transmission ${T}_{m}\left(\theta ,\varphi \right)$. In the third step, the measured transmission obtained was compared with the simulated transmission ${T}_{s}\left(\theta ,\varphi , \overline{\rho }\right)$ to infer the average density values along the LoS of the detector (density map). Therefore, these ${T}_{s}$ values depend on the constant average density assigned to the rock above the detector in the simulation. For each chosen density, the simulated transmission was compared with the measured one to obtain the density profile values along the LoS $\left(\theta ,\varphi \right)$ of the muon tracker in the target configuration. This third step strictly depends on the differential muon flux model used for the Earth surface and on the three-dimensional geometry of the studied target, that is, the three-dimensional model resulting from the applied RS methods or from synthetic geometrical modeling. An important feature is the angular resolution of the tracker used (Table 1), which allows us to solve objects with dimensions compatible with the cavities we are looking for. Quantitatively, considering that the achievable resolution depends on the distance between the unknown cavity and the center of the detector, we potentially expect cavities (tombs) at a distance from the detector ranging from 20 to 100 m. Therefore, using the MIMA detector, that has an angular resolution of about 7 mrad, we could potentially solve up to 0.14 and 0.70 m, respectively. This detection limit is sufficient for identifying potential tombs within the studied target, i.e. the eastern hill of the Palazzone necropolis. The tombs dimensions (length, width, or height) should vary between 2 and 5 m, with volumes ranging between 5 and 30 m^3^. According to archaeologists, the planned muography survey aims to detect potentially undiscovered single-chamber tombs and/or the remains of larger tombs (collapsed chambers). We refer to lower-volume cavities (< 30 m^3^) in comparison with those already discovered and inventoried.

Because the objective of this paper is to show the importance of the RS survey methods in the muon imaging process, the authors have decided not to go deeper into the details of the muon imaging process and the MIMA detector itself. Detailed descriptions of these topics and explanations of the muon imaging process, and reconstruction of 2D/3D density anomalies, have been reported in more specific published papers^27,36,37,40,54,77–79^.

## Conclusion

This paper describes how the accuracy of the three-dimensional model of the reconstructed ground surface of the investigated target can enhance or worsen muon imaging results. The muographic measurement campaign at the Palazzone necropolis enabled us to understand, quantify and maximize the contribution of remote sensing (RS) outputs for muon imaging purposes (depth map analysis).

The use of different RS methods, particularly close-range ones, allowed us to create the most accurate three-dimensional model of the studied target. By means of three geomatic survey methods, namely ALS, TLS and UAV-DP techniques, we have created a unique three-dimensional environment of the northeast side of the Palazzone necropolis (the target hill) characterized by three possible resolution values: 1 m (ALS survey), 0.2 m (UAV-DP) and 0.02 m (TLS). This merged model has proven to be more reliable than only ministerial 1 × 1 m^2^ ALS or 10 × 10 m^2^.

The final accuracy achieved during the three-dimensional ground surface reconstruction of the studied hill directly influenced the muography measurement, and therefore, the possibility of obtaining reliable results in terms of simulated transmission and density maps.

These results confirm that high-resolution three-dimensional data can contribute to reliable muographic data simulations, analyses and outputs. Throughout the proposed workflow, the authors stressed that it is fundamental to consider potential errors arising from inaccurate topographical data because of the complex natural environment (geomorphology and vegetation issues) or inaccurate geomatic surveys. This is particularly true for archaeological applications of muography because the density anomalies being sought are of the same magnitude as the potential inaccuracies for some $\left(\theta ,\varphi \right)$. As in the case of the Palazzone necropolis, some angular sectors of the MIMA acceptance, primarily those where the MIMA LoS are parallel to the dip direction of the hill slopes, can present thickness errors along the LoS greater than 5 m and up to 20 m (see depth maps in Fig. 9), which roughly correspond to the dimensions of potential unknown tombs excavated on the slope. To prevent thickness inaccuracies induced by the aforementioned causes from affecting the final density map created by muography, the authors suggest discarding the angular area most sensitive to potential ground surface errors (Fig. 9c-e) or, if it is worth, considering these angular regions by taking into account the estimated errors on *L*. In addition, the obtained results imply that in a scenario comparable to the one provided, the contributions of probable elevation errors cannot be ignored and should be quantified in the same manner as those generally evaluated.

The results of this study highlight the importance of TLS and UAV-DP techniques as support tools for making hypotheses, decisions and analysis during muographic measurement campaigns, confirming how the integrated use of different RS methods may aid in achieving reliable density maps in muography applications, thereby confirming the multidisciplinary identity of muon radiography.

## Data Availability

The datasets used and/or analysed during the current study are available from the corresponding author on reasonable request.

## References

[1] Telling J, Lyda A, Hartzell P, Glennie C (2017). Review of Earth science research using terrestrial laser scanning. Earth-Sci. Rev..

[2] Sturzenegger M, Stead D (2009). Close-range terrestrial digital photogrammetry and terrestrial laser scanning for discontinuity characterization on rock cuts. Eng. Geol..

[3] Peter Heng BC, Chandler JH, Armstrong A (2010). Applying close range digital photogrammetry in soil erosion studies: Applying close range digital photogrammetry in soil erosion studies. Photogramm. Rec..

[4] Westoby MJ, Brasington J, Glasser NF, Hambrey MJ, Reynolds JM (2012). ‘Structure-from-Motion’ photogrammetry: A low-cost, effective tool for geoscience applications. Geomorphology.

[5] Jaboyedoff M (2012). Use of LIDAR in landslide investigations: a review. Nat. Hazards.

[6] Radicioni F, Stoppini A, Tosi G, Marconi L (2021). Necropolis of Palazzone in Perugia: Geomatic data integration for 3D modeling and geomorphology of underground sites. Trans. GIS.

[7] Abellán A (2014). Terrestrial laser scanning of rock slope instabilities. Earth Surf. Process. Landf..

[8] Stead D, Donati D, Wolter A, Sturzenegger M (2019). Application of remote sensing to the investigation of rock slopes: Experience gained and lessons learned. ISPRS Int. J. Geo-Inf..

[9] Rhee DS, Kim YD, Kang B, Kim D (2018). Applications of unmanned aerial vehicles in fluvial remote sensing: An overview of recent achievements. KSCE J. Civ. Eng..

[10] *3D Recording and Modelling in Archaeology and Cultural Heritage: Theory and Best Practices*. (Archaeopress, 2014).

[11] Vasuki Y, Holden E-J, Kovesi P, Micklethwaite S (2014). Semi-automatic mapping of geological structures using UAV-based photogrammetric data: An image analysis approach. Comput. Geosci..

[12] Bertacchi A (2022). UAVs technology as a complementary tool in post-fire vegetation recovery surveys in Mediterranean fire-prone forests. Forests.

[13] Lague D, Brodu N, Leroux J (2013). Accurate 3D comparison of complex topography with terrestrial laser scanner: Application to the Rangitikei canyon (N-Z). ISPRS J. Photogramm. Remote Sens..

[14] Gigli G, Morelli S, Fornera S, Casagli N (2014). Terrestrial laser scanner and geomechanical surveys for the rapid evaluation of rock fall susceptibility scenarios. Landslides.

[15] Ahmad Fuad, N., Yusoff, A. R., Ismail, Z. & Majid, Z. Comparing the performance of point cloud registration methods for landslide monitoring using mobile laser scanning data. *Int. Arch. Photogramm. Remote Sens. Spat. Inf. Sci.* **XLII-4/W9**, 11–21 (2018).

[16] Battulwar R, Zare-Naghadehi M, Emami E, Sattarvand J (2021). A state-of-the-art review of automated extraction of rock mass discontinuity characteristics using three-dimensional surface models. J. Rock Mech. Geotech. Eng..

[17] Tucci, G., Bonora, V., Conti, A. & Fiorini, L. High-quality 3D models and their usein a cultural heritage conservation project. *Int. Arch. Photogramm. Remote Sens. Spat. Inf. Sci.* **XLII-2/W5**, 687–693 (2017).

[18] Lercari N (2019). Monitoring earthen archaeological heritage using multi-temporal terrestrial laser scanning and surface change detection. J. Cult. Herit..

[19] Flener C (2013). Seamless mapping of river channels at high resolution using mobile LiDAR and UAV-photography. Remote Sens..

[20] Xu Z (2014). Tridimensional reconstruction applied to cultural heritage with the use of camera-equipped UAV and terrestrial laser scanner. Remote Sens..

[21] Rossi G (2018). Multitemporal UAV surveys for landslide mapping and characterization. Landslides.

[22] Bonechi L, D’Alessandro R, Giammanco A (2020). Atmospheric muons as an imaging tool. Rev. Phys..

[23] Cimmino L (2021). Principles and perspectives of radiographic imaging with muons. J. Imaging.

[24] Zyla, P. A. *et al.* Review of Particle Physics. *Prog. Theor. Exp. Phys.* **2020**, 083C01 (2020).

[25] Tanaka HKM, Kusagaya T, Shinohara H (2014). Radiographic visualization of magma dynamics in an erupting volcano. Nat. Commun..

[26] Baccani G (2019). Muon radiography of ancient mines: The San Silvestro Archaeo-Mining Park (Campiglia Marittima, Tuscany). Universe.

[27] Saracino G (2019). Applications of muon absorption radiography to the fields of archaeology and civil engineering. Philos. Trans. R. Soc. Math. Phys. Eng. Sci..

[28] Nishiyama R (2017). First measurement of ice-bedrock interface of alpine glaciers by cosmic muon radiography. Geophys. Res. Lett..

[29] Athanassas CD (2020). Muography for geological hazard assessment in the South Aegean active volcanic arc (SAAVA). Mediterr. Geosci. Rev..

[30] Tanaka HKM (2016). Instant snapshot of the internal structure of Unzen lava dome, Japan with airborne muography. Sci. Rep..

[31] Marteau J (2017). DIAPHANE: Muon tomography applied to volcanoes, civil engineering, archaelogy. J. Instrum..

[32] Tioukov V (2019). First muography of Stromboli volcano. Sci. Rep..

[33] Bryman, D. *et al.* Muon geotomography—Bringing new physics to orebody imaging. In *Building Exploration Capability for the 21st Century* (Society of Economic Geologists, 2014). 10.5382/SP.18.11.

[34] Schouten D, Ledru P (2018). Muon tomography applied to a dense uranium deposit at the McArthur River Mine. J. Geophys. Res. Solid Earth.

[35] Zhang Z-X, Enqvist T, Holma M, Kuusiniemi P (2020). Muography and its potential applications to mining and rock engineering. Rock Mech. Rock Eng..

[36] Beni T (2023). Transmission-based muography for ore bodies prospecting: A case study from a Skarn Complex in Italy. Nat. Resour. Res..

[37] Baccani G (2021). The reliability of muography applied in the detection of the animal burrows within River Levees validated by means of geophysical techniques. J. Appl. Geophys..

[38] Alvarez LW (1970). Search for hidden chambers in the pyramids: The structure of the second pyramid of Giza is determined by cosmic-ray absorption. Science.

[39] Morishima K (2017). Discovery of a big void in Khufu’s Pyramid by observation of cosmic-ray muons. Nature.

[40] Borselli D (2022). Three-dimensional muon imaging of cavities inside the Temperino mine (Italy). Sci. Rep..

[41] Procureur S (2023). Precise characterization of a corridor-shaped structure in Khufu’s Pyramid by observation of cosmic-ray muons. Nat. Commun..

[42] Caffau, E., Coren, F. & Giannini, G. Underground cosmic-ray measurement for morphological reconstruction of the “Grotta Gigante” natural cave. *Nucl. Instrum. Methods Phys. Res. Sect. Accel. Spectrometers Detect. Assoc. Equip.* **385**, 480–488 (1997).

[43] Nishiyama R, Miyamoto S, Okubo S, Oshima H, Maekawa T (2017). 3D density modeling with gravity and muon-radiographic observations in Showa-Shinzan Lava Dome, Usu, Japan. Pure Appl. Geophys..

[44] Lesparre N (2012). Density muon radiography of La Soufrière of Guadeloupe volcano: Comparison with geological, electrical resistivity and gravity data. Geophys. J. Int..

[45] Oláh L (2023). Muon imaging of volcanic conduit explains link between eruption frequency and ground deformation. Geophys. Res. Lett..

[46] Schouten D (2019). Muon geotomography: selected case studies. Philos. Trans. R. Soc. Math. Phys. Eng. Sci..

[47] Procureur, S. Muon imaging: Principles, technologies and applications. *Nucl. Instrum. Methods Phys. Res. Sect. Accel. Spectrometers Detect. Assoc. Equip.* **878**, 169–179 (2018).

[48] Kaiser R (2019). Muography: Overview and future directions. Philos. Trans. R. Soc. Math. Phys. Eng. Sci..

[49] Lechmann A (2021). Muon tomography in geoscientific research—A guide to best practice. Earth-Sci. Rev..

[50] QGIS.org. *QGIS Geographic Information System Version 3.24.0. QGIS Association*. http://www.qgis.org. *The Used Satellite Base Map*. http://www.google.cn/maps/vt?lyrs=s@189&gl=cn&x={x}&y={y}&z={z} (2023).

[51] Melelli L, Bizzarri R, Baldanza A, Gregori L (2016). The Etruscan “Volumni Hypogeum” Archeo-Geosite: New sedimentological and geomorphological insights on the Tombal Complex. Geoheritage.

[52] Silene. *Progetto Silene*. http://silenepg.it/ (2023).

[53] Tanaka HKM (2007). Imaging the conduit size of the dome with cosmic-ray muons: The structure beneath Showa-Shinzan Lava Dome, Japan. Geophys. Res. Lett..

[54] Saracino, G. *et al.* Imaging of underground cavities with cosmic-ray muons from observations at Mt. Echia (Naples). *Sci. Rep.* **7**, 1181 (2017). 10.1038/s41598-017-01277-3 PMC5430851 28446789

[55] Nishiyama R (2019). Bedrock sculpting under an active alpine glacier revealed from cosmic-ray muon radiography. Sci. Rep..

[56] Bonechi, L. *et al.* Development of the ADAMO detector: Test with cosmic rays at different zenith angles. In *29th International Cosmic Ray Conference (Pune)*. Vol. 9. 283–286 (2005).

[57] Geoportale Nazionale. *DTM Lidar 1 Meter Ground Resolution, Umbria Region.* http://www.pcn.minambiente.it/viewer/index.php?services=LiDAR_Umbria (2023).

[58] CloudCompare. *CloudCompare*. https://www.danielgm.net/cc/ (2022).

[59] Brodu N, Lague D (2012). 3D terrestrial lidar data classification of complex natural scenes using a multi-scale dimensionality criterion: Applications in geomorphology. ISPRS J. Photogramm. Remote Sens..

[60] Martorana R, Capizzi P, Pisciotta A, Scudero S, Bottari C (2023). An overview of geophysical techniques and their potential suitability for archaeological studies. Heritage.

[61] Tioukov V (2017). Muography with nuclear emulsions—Stromboli and other projects. Ann. Geophys..

[62] Holma, M., Zhang, Z., Kuusiniemi, P., Loo, K. & Enqvist, T. Future prospects of muography for geological research and geotechnical and mining engineering. In *Geophysical Monograph Series* (eds. Oláh, L., Tanaka, H. K. M. & Varga, D.). 199–219 (Wiley, 2022). 10.1002/9781119722748.ch15.

[63] Liu G (2023). High-precision muography in archaeogeophysics: A case study on Xi’an defensive walls. J. Appl. Phys..

[64] Malmqvist L, Jönsson G, Kristiansson K, Jacobsson L (1979). Theoretical studies of in-situ rock density determinations using underground cosmic-ray muon intensity measurements with application in mining geophysics. Geophysics.

[65] Gómez H (2017). Forward scattering effects on muon imaging. J. Instrum..

[66] Zhang J-M (2023). Influence of multiple Coulomb scattering on accuracy of muon transmission imaging of small-scale matter. Acta Phys. Sin..

[67] Guardincerri E (2017). 3D cosmic ray muon tomography from an underground tunnel. Pure Appl. Geophys..

[68] Riegl. *Riegl VZ-1000 Datasheet*. (2023).

[69] Weidner L, Walton G, Krajnovich A (2021). Classifying rock slope materials in photogrammetric point clouds using robust color and geometric features. ISPRS J. Photogramm. Remote Sens..

[70] Giordan D (2020). The use of unmanned aerial vehicles (UAVs) for engineering geology applications. Bull. Eng. Geol. Environ..

[71] Pix4D. *Pix4Dmapper Software*. https://www.pix4d.com/product/pix4dmapper-photogrammetry-software (2022).

[72] Agisoft. *Agisoft Metashape Software*. https://www.agisoft.com/ (2023).

[73] Baccani, G. *et al.**The MIMA Project. Design, Construction and Performances of a Compact Hodoscope for Muon Radiography Applications in the Context of Archaeology and Geophysical Prospections*. (2018). 10.48550/ARXIV.1806.11398.

[74] Cimmino L (2017). The MURAVES telescope front-end electronics and data acquisition. Ann. Geophys..

[75] Cimmino L (2019). 3D muography for the search of hidden cavities. Sci. Rep..

[76] Bonechi L (2020). Multidisciplinary applications of muon radiography using the MIMA detector. J. Instrum..

[77] Viliani L (2012). Muon Radiography of Underground Structures with an Odoscope: Feasibility Study and Early Developments.

[78] Bonechi L (2018). The MURAVES project and other parallel activities on muon absorption radiography. EPJ Web Conf..

[79] Bonechi L (2019). Tests of a novel imaging algorithm to localize hidden objects or cavities with muon radiography. Philos. Trans. R. Soc. Math. Phys. Eng. Sci..

